# Evaluation of Power Production Asymmetry during Cycling in Persons with Multiple Sclerosis

**DOI:** 10.3390/ijerph16183445

**Published:** 2019-09-17

**Authors:** John W. Farrell, Debra A. Bemben, Christopher D. Black, Daniel J. Larson, Gabriel Pardo, Cecilie Fjeldstad-Pardo, Rebecca D. Larson

**Affiliations:** 1 Interdisciplinary School of Health Science, University of Ottawa, Ottawa, ON K1N 6N5, Canada; 2 Department of Health and Exercise Science, University of Oklahoma, Norman, OK 73079, USA; dbemben@ou.edu (D.A.B.); cblack@ou.edu (C.D.B.); larsondj@ou.edu (D.J.L.); rdlarson@ou.edu (R.D.L.); 3 Multiple Sclerosis Center of Excellence, Oklahoma Medical Research Foundation, Oklahoma City, OK 73104, USA; gabriel-pardo@omrf.org (G.P.); Cecilie-Fjeldstad@omrf.org (C.F.-P.)

**Keywords:** multiple sclerosis, asymmetry, exercise, lower extremity, walking

## Abstract

Lower limb asymmetries have been observed in persons with multiple sclerosis (PwMS), and have been associated with mobility impairment. An incremental cycling test was performed on a cycle ergometer to determine peak power output (PPO) and peak oxygen consumption (VO_2_peak). Then, participants cycled at 50%, 60%, and 70% of their PPO to assess the contribution of each lower limb to power production. Two-way repeated measures ANOVA was used to detect group × intensity differences in power production asymmetry. Eight PwMS and six healthy individuals (Non-MS) completed the study. No statistically significant (*p* > 0.05) group × intensity interactions or main effects were present when examining between-limb differences in power production. The current data do not indicate a statistically significant difference in power production asymmetry between groups and exercise intensities. Previous research has established a 10% difference between contralateral limbs as a threshold for asymmetry. The average asymmetry in power production in PwMS exceeded the 10% threshold at all measured outputs, suggesting the presence of asymmetry in power production.

## 1. Introduction

Multiple sclerosis is a chronic neurological disease characterized by disruption in the propagation of action potentials along the axons of neurons due to the demyelination of the myelin sheath [1]. The formation of scleroses, or plaques, in the white and gray matter of the brain and spinal cord has been associated with negative alterations in the peripheral muscular tissue that includes: reductions in enzyme oxidative capacity, impairment of the excitation–contraction coupling processes, and atrophy [2,3,4,5,6,7,8]. Due to these alterations in muscle function and performance, persons with multiple sclerosis (PwMS) can experience a lower exercise tolerance, an increase in the perceived difficulty to perform activities of daily living, and a reduction in quality of life [9,10].

Previous observations suggest that PwMS may experience decrements in muscle function in an asymmetrical pattern [3,11,12,13]. A greater decrement in muscle function is observed in a limb compared to the contralateral limb, creating an imbalance between sides of the body. Lower limb asymmetry has been observed in PwMS for: muscular strength, oxygen uptake, femoral neck bone mineral density, and work performed and power output during unilateral incremental cycling [3,11,12,13]. Significant associations have been observed between lower limb asymmetries and MS-related fatigue, although other investigations have reported no associations [3,14]. Additionally, lower limb asymmetries have been associated with walking impairment and postural instability [3,13]. Future investigations are required to further explore the potential negative effects of lower limb asymmetries on functional capacity and MS-related symptoms.

Previous studies have assessed asymmetry by isolating the lower limbs and requiring each limb to work independently of the other (i.e., unilateral cycling) [11,12,13]. Although this methodology has provided valuable insight, it has not allowed for an assessment of how lower limb asymmetry may influence natural bipedal movements and exercises, highlighting an area of research that needs to be addressed. Additionally, previous research has predominately assessed asymmetry at maximal intensities, with only one study assessing asymmetry in PwMS during unilateral cycling at low levels of exercise intensity (20% of whole body peak workload) [11,12,13]. The physical manifestation of asymmetry as being intensity dependent has yet to be evaluated in PwMS.

Extensive and consistent data is available to support the beneficial effects of exercise training on muscular strength, aerobic capacity, and ambulatory performance in PwMS [15]. However, based on previously reported associations, lower limb asymmetry may negatively impact PwMS’ ability to safely participate in rehabilitation programs and exercise training. Lower limb asymmetry has not previously been considered for exercise prescription in PwMS, potentially placing PwMS at increased risk for falls during exercise. To better understand the impact of lower limb asymmetry on exercise capacity in PwMS, assessing asymmetry during a natural bipedal movement in addition to assessing the impact of exercise intensity on the manifestation of asymmetry is required. The integration of dual power meters into cycle ergometers now allows for simultaneous limb assessment and the quantification of each limb’s contribution to total power output during cycling while allowing for the precise control of intensity in an exercise modality that is conducive for those who may be at risk for falls. Therefore, the aims of the current study were to: (1) investigate whether PwMS exhibit greater lower limb asymmetry in force application and power production contribution during double-leg cycling compared to healthy controls, and (2) investigate how exercise intensity affects the physical manifestation of lower limb asymmetry in PwMS. We hypothesized that (1) PwMS would exhibit a greater lower limb asymmetry in force production and power production contribution during cycling compared to healthy controls, and (2) that asymmetry in PwMS would be greatest at higher exercise intensities. This study will provide new insight on the presentation and extent of lower limb asymmetry in PwMS, which can inform the design and prescription of rehabilitation programs and exercise training.

## 2. Materials and Methods

Research Design: The current study utilized a mixed factorial design and consisted of 4 laboratory visits with a minimum of 48 h in between. All subjects gave their informed consent for inclusion before they participated in the study. The study was conducted in accordance with the Declaration of Helsinki, and the protocol was approved by the Institutional Review Board of the University of Oklahoma (IRB number 8069). Visit 1 consisted of participants being familiarized with all equipment and testing protocols prior to testing, and conducting a body composition assessment via a dual-energy X-ray absorptiometry scan. During Visit 2, participants performed an incremental cycling test (ICT) to task failure at a self-selected cadence to determine peak oxygen consumption (VO_2_peak) and peak power output (PPO). Upon completion of the ICT, participants were allotted 20 minutes (min) of rest before completing a verification protocol to validate the VO_2_peak and PPO determined from the ICT. On Visit 3, participants completed a submaximal ICT that included stages at 50%, 60%, and 70% of PPO. Walking capacity was assessed via the 6-Minute Walk Test (6MWT) and Timed 25-Foot Walk (T25FW) during Visit 4 in addition to knee extensor maximal voluntary isometric strength. Both the Modified Fatigue Impact Scale (MFIS) and Rochester Fatigue Diaries (RFD) were assessed prior to each testing session to ensure consistent levels of fatigue across sessions [16,17]. Testing sessions were rescheduled to a later date if an MFIS score greater than 2.5 standard deviations from previous scores was recorded, or if upon the visual inspection of RFDs, elevated fatigue levels were present [16,17].

Participants: The current sample consisted of 14 participants, including: 8 persons with MS (3 females and 5 males), and 6 healthy persons without MS (3 females and 3 males) matched by age, height, weight, and gender. Persons with MS were required to have a confirmed diagnosis of relapsing–remitting MS from a physician, an Expanded Disability Status Scale (EDSS) score of ≤6.0 (minimal to moderate disability—may need intermittent or unilateral aid to walk 100 m), and be free from a relapse for the 3 months prior to testing. A relapse was defined as a period of worsening symptoms lasting longer than 24 h. Participants with any previous or current lower limb orthopedic alignments or procedures (arthritis, hip replacement, knee surgery, etc.) were excluded. Participants using prednisone or who had a steroid dose less than three months prior to testing were excluded.

Body Composition: Lower-limb composition was assessed using a whole-body Lunar dual-energy X-ray absorptiometry scanner (with software version 13.60.033, GE-Lunar Prodigy Advanced, Madison, WI, USA). Pre-scan calibration quality assurance indicated a low coefficient of variation (<0.2%). Subjects were positioned in the center of the dual-energy X-ray absorptiometry table in the supine position using standardized positioning; the arms were close to the sides of the body, and the legs were secured by Velcro straps to ensure proper positioning and spacing between limbs for segmental analysis. Subjects too wide for the scanning bed had each side of the body tested separately, and the composition of both sides of the body were added together to estimate body composition. Assessment of the lower limbs was used to determine any significant differences in the lean mass of the legs between groups. From the full-body scans, separate regions of interest were made of the lower legs, using the tibiofemoral joint of the knee and subtalar joint of the ankle as landmarks. The region of interest for each lower limb was quality checked by two separate researchers to ensure accuracy.

Incremental Cycling Test: A magnetically braked cycle ergometer (Sport Excalibur, Lode; B.V. Medical Technology, Groningen, The Netherlands) along with a metabolic cart (True One 2400, Parvo Medics, Sandy, UT, USA) was utilized to perform all incremental cycling tests (ICTs). Subjects were instructed to abstain from exercise and caffeine 12 h prior to testing and to fast three to four hours prior to testing. Subjects were instructed to pedal at a cadence that was comfortable and they felt could be maintained for an extended period of time. Following a one-minute rest period and a five-minute warm up at 50 watts (W), the graded exercise test was initiated at a workload of 1.0 W per kilogram of body mass (W × BWkg^−1^) and increased by 0.5 W × BWkg^−1^ every three minutes until the participant reached task failure, as indicated by a pedal rate dropping more than 10 revolutions per minute from their self-selected cadence [18]. Heart rate (HR) was measured via a telemetric HR monitor (Polar T31, Polar Electro Inc., Bethpage, NY, USA) throughout the graded exercise test, and rating of perceived exertion (RPE) was measured at the end of each three-minute stage [19]. Metabolic and ventilatory data were continuously measured and averaged over 30-s intervals. VO_2_peak was indicated by the highest 30-s average recorded during the final stage of the test.

Verification: VO_2_peak was verified using the protocol developed by Nolan et al. [20]. Participants were given 20 min of rest between the completion of the ICT and before beginning the verification protocol. Using the PPO obtained during the ICT, participants performed a multistage warm-up that consisted of 2 min at 50% of PPO followed by 1 min at 70% of PPO. Then, the workload increased to 105% of PPO, and participants were instructed to maintain their self-selected cadence for as long as possible. When cadence decreased by greater than 10 revolutions per minute, exercise was terminated [20]. VO_2_peak was determined as the highest 30-s average obtained from either the initial ICT or the verification protocol.

Submaximal ICT: Using the PPO and VO_2_peak collected from the ICT and verification protocol, the subsequent submaximal ICT was designed in a manner that allowed for participants to exercise at specific relative exercise intensities. The testing consisted of a 3-min warm-up at 25% of PPO followed by 3-min stages at 50%, 60%, and 70% of the individual’s PPO. Metabolic and ventilatory data were continuously measured and averaged over 30-s intervals. HR was measured throughout each test, with RPE measured at the end of each stage. [19]. Power (measured in W) and force (indicated by peak torque (N·m)) from each limb was collected during each stage to assess the presence of asymmetries. Additionally, mechanical efficiency was assessed during each stage and defined as the percent of power produced during the crank arm cycle that translates to forces generating forward propulsion, with higher values being indicative of greater efficiency.

Lower Limb Strength: Maximal voluntary isometric contractions (MVCs) of the knee extensors were assessed using a dynamometer (KinCom model: KC125AP, Isokinetic International, East Ridge, TN, USA). Subjects were seated with hip and knee angle set at 70°. Participants were asked to perform a series of warm-up isometric contractions at submaximal intensities with 2 to 3 min of rest between contractions. Following the warm-up, participants performed 3 MVCs lasting 3 s each, with 3 min of rest between contractions. Both legs were assessed, and the order was randomly selected. The two highest values for each participant were averaged.

Walking Capacity: Both the T25FW and 6MWT are assessment tools utilized by both researchers and clinicians to evaluate disease progression and walking capacity in PwMS [21,22]. The T25FW requires participants to begin in a standing position and walk 25 feet as quickly as possible, but safely [21]. The participants were instructed to walk back to the starting point, and the test was performed again. The dependent variable was the average amount of time (seconds) required to walk 25 feet over the two trials [21]. The 6MWT was conducted on a 60-m marked course, and participants were instructed to cover as much ground as possible in 6 min while walking [22]. The total distance covered was recorded to the nearest meter [22].

Asymmetry Scores: Scores were assigned to strength measurements collected during the MVCs. The equation below was used, with 0% indicating an even distribution of strength across the limbs, and 100% indicating maximal asymmetry [3].

$$Strength\;asymmetry\;score=\;\left[1-\left(\frac{Power\;of\;Weaker\;Limb}{Power\;of\;Stronger\;Limb}\right)\right]100$$

The assignment of a stronger and weaker limb, in terms of power or torque production, was not possible during the submaximal ICT. This was due to the lack of a consistent pattern of dominance in one leg for force application and power production throughout the submaximal ICT. Limb preference has been noted to change during bilateral movements depending on the complexity and conditions during the movement [13,23]. For this reason, asymmetry values during the submaximal graded exercise test were calculated as the absolute difference between the limbs (|left leg − right leg|), with higher values indicating greater differences between limbs. Additionally, the percent difference between contralateral limbs in muscle function has been previously used to detect asymmetry, with a threshold of ≥10% being used to indicate asymmetry being present [14,24,25]. This method was used in addition to statistical analysis to detect asymmetry.

Statistical Analysis: All analyses were performed using IBM SPSS Statistics (version 25.0; IBM Corp., Armonk, NY, USA). Descriptive statistics were used to summarize the demographic data. A t-test analysis of independent samples using difference scores was used to assess differences in lower limb body composition between groups. T-tests on independent samples were used to assess isometric strength asymmetry and walking capacity during functional performance tests between groups. Two-way repeated measures analysis of variance (ANOVA) were used to detect group × intensity interactions for the power, force, and physiological variables collected during the submaximal ICTs. When significant interactions and effects were found, Bonferroni corrections were performed for post hoc analysis. An alpha level of ≤0.05 was the criteria to establish statistically significant differences. Cohen’s d effect sizes were analyzed when appropriate, with values of 0.2, 0.5, and 0.8 indicating small, moderate, and large effects, respectively [26]. Effect sizes for ANOVA were analyzed when appropriate using eta-squared (η^2^). A value of 0.02 was considered a small effect, 0.13 was considered a medium effect, and 0.26 was considered a large effect [26,27]. Bivariate Spearman correlations were used to examine potential associations between asymmetries and walking performance. Correlation coefficient values of 0.1, 0.3, and 0.5 were interpreted as small, moderate, and large, respectively [26].

## 3. Results

Participant Characteristics: Fourteen individuals completed the study and were included in data analysis. Five males and 3 females (*n* = 8) were included in the PwMS group, and 3 males and 3 females (*n* = 6) were included in the Non-MS group. Descriptive and anthropometric data for both groups are listed in Table 1. There were no significant between-group differences (*p* > 0.05) for all descriptive and anthropometric variables. All the PwMS possessed a physician’s diagnosis of relapse remitting MS, and had an average EDSS score of 1.87 ± 1.09 indicating a minimal impairment in a neurological category. One visit for one subject was rescheduled to a later date due to elevated levels of fatigue as assessed via the RFD and MFIS prior to the testing session.

Body Composition: Using the lower limb classifications (i.e., strong and weak) to determine strength asymmetry scores, the differences (strong–weak) in lean and fat mass for lower limbs are presented for each group in Table 2. The results of the independent t-test indicated no significant differences between groups (*p* > 0.05) for between-limb differences in the lean mass, fat mass, and fat percentage of the lower limbs with small, small, and large effect sizes observed, respectively.

Initial ICT: Physiological variables collected during the initial ICT are reported in Table 3. No significant differences (*p* > 0.05) between groups were observed, with small effect sizes for all variables.

**Figure 1:**
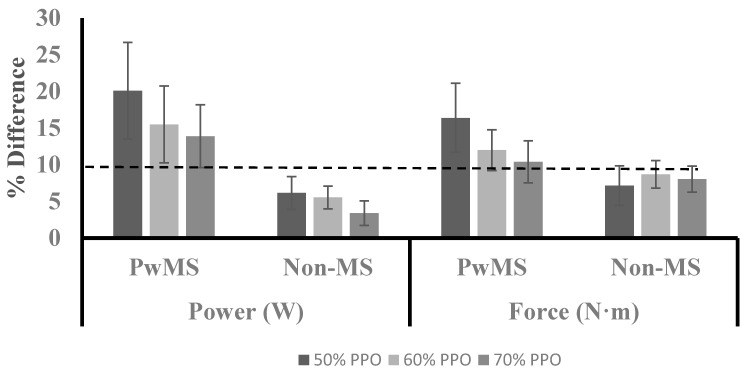
Average lower limb asymmetry in power and force. Data are presented as mean ± standard error. Dotted line indicates 10% threshold for asymmetry.

Submaximal ICT Variables: During submaximal ICTs, both the power and force for each leg were assessed in addition to efficiency. The results are reported in Table 4 for both absolute and percent differences in power and force between limbs and efficiency during each stage. No statistically significant group × intensity interactions or main effects were present when examining the absolute and percent differences in power and force. However, a main effect was present for intensity when examining efficiency (F = 99.2, *p* = 0.00, η^2^ = 0.948). Post hoc analysis indicated that when collapsed across groups, efficiency significantly increased as intensity increased. Figure 1 illustrates the average percent difference in power and force for both groups across each stage. A large range of values were observed for both power (range at 50%: 0.70 to 43.9; 60%: 0.10 to 37.3; 70%: 0.58 to 32.4) and force (range at 50%: 0.09 to 38.9; 60%: 2.69 to 26.5; 70%: 0.95 to 25.2) in PwMS, while a smaller range of values was observed in the Non-MS group for power (range at 50%: 0.38 to 13.1; 60%: 1.78 to 10.8; 70%: 0.12 to 10.7) and force (range at 50%: 0.31 to 18.2; 60%: 3.12 to 14.5; 70%: 1.53 to 13.9).

**Figure 2:**
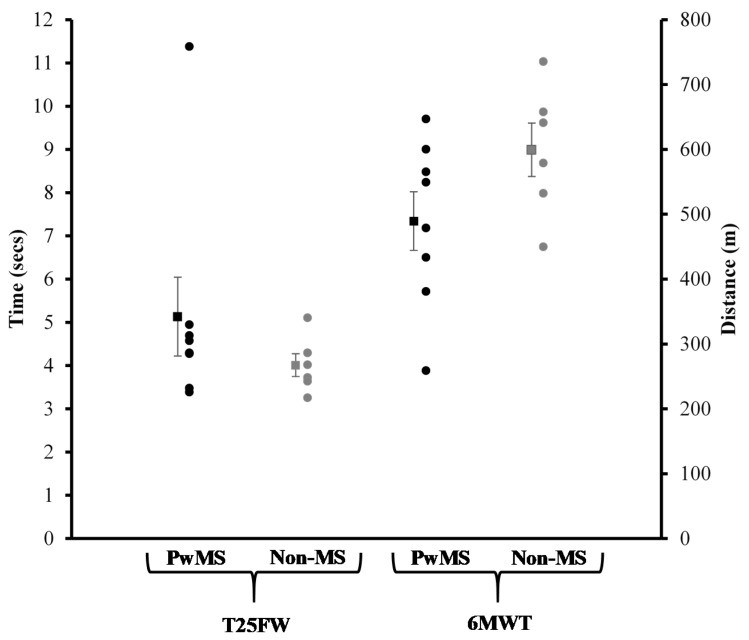
Walking capacity assessments. Data are presented as mean ± SE.

**Figure 3:**
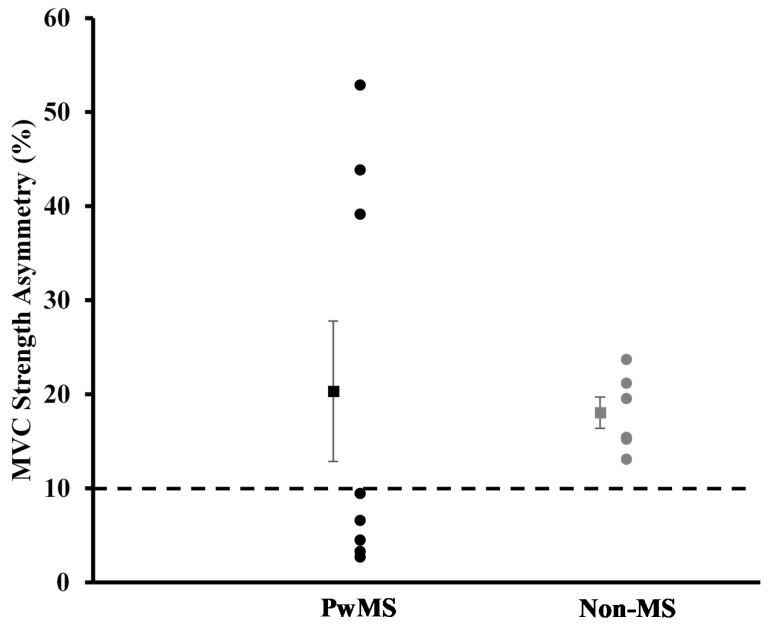
Maximal voluntary isometric contractions (MVC) strength asymmetry score. Data are presented as mean ± SE. Dotted line indicates 10% threshold for asymmetry.

Walking Capacity and MVCs: Individual results and group averages for performance on the T25FW test and 6MWT are displayed in Figure 2. Figure 3 represents individual results and the group average for knee extensor strength asymmetry scores. No statistically significant differences between groups were detected for performance on the T25FW, 6MWT, and knee extensor strength asymmetry scores (*p* > 0.05).

**Figure 4:**
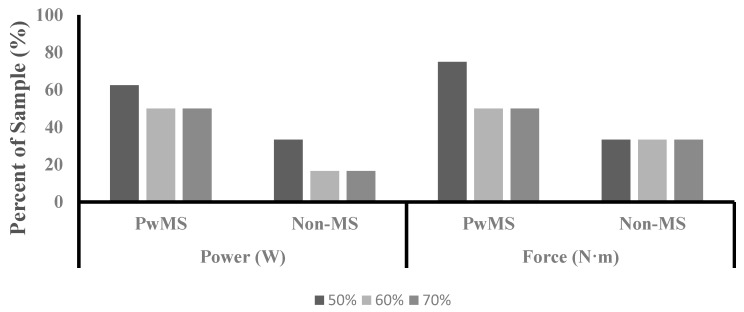
Percent of sample that exhibited ≥10% asymmetry. Data displayed as frequency data.

Frequency of Asymmetry: Figure 4 illustrates the percent of the sample for each group that exhibited ≥10% asymmetry for power and force.

Correlation analysis: Correlation analysis was performed to determine the association between variables collected during the submaximal ICTs and walking performance (T25FW and 6MWT) (Table 5). No significant correlations between physiological variables and walking capacity tests were present in PwMS (*p* > 0.05). However, the Non-MS group did display a significant correlation between performance on the T25FW and efficiency at 50% (*p* = 0.05, r = −0.812), 60% (*p* = 0.04, r = −0.833), and 70% (*p* = 0.24, r = −0.870) PPO, as well as a percent difference in force at 70% PPO (*p* = 0.03, r = 0.844). Additionally, no significant associations were present between knee extensor strength asymmetry scores and performance on T25FW and 6MWT (*p* > 0.05) (Table 6).

## 4. Discussion

The current study examined the effects of exercise intensity on the physical manifestation of lower limb asymmetry when exercising in a bipedal movement in PwMS. We hypothesized that PwMS would exhibit greater levels of asymmetry in power and force production compared to Non-MS participants. However, our results indicate that no statistically significant differences were present between the groups for levels of asymmetry in force application power and production; thus, we rejected our hypothesis. Additionally, we hypothesized that exercise intensity would have a significant effect on the level of asymmetry in power and force production in PwMS, with higher exercise intensities eliciting greater levels of asymmetry. Our results show that exercise intensity did not have a significant effect on levels of asymmetry in PwMS; thus, we rejected our hypothesis. It was revealed that a main effect was present for exercise intensity on efficiency, indicating greater levels of efficiency at higher exercise intensities.

Statistically significant lower limb asymmetry in PwMS has been reported previously for VO_2_peak, PPO, and work performed during unilateral cycling [11,12,13]. However, the current study did not observe statistically significant levels of asymmetry in PwMS. The contrasting findings between the current study and that of the previous literature could potentially be due to methodological differences in the assessment of asymmetry. Previous investigations have been limited to assessing each limb individually during unilateral cycling, while the current study was able to assess both limbs simultaneously during bipedal cycling [11,12,13]. Compared to traditional bipedal cycling, unilateral cycling may potentially require the activation of different muscles, or greater activation of the same muscles at different points throughout the crank arm cycle resulting in a distinct metabolic response. Additionally, the exercise intensity in which lower limb asymmetry was assessed differed between the current study and previous literature. Previously, asymmetry in power production during cycling has only been assessed at intensities equal to PPO and 20% of PPO during unilateral cycling, while the current study assessed asymmetry at 50%, 60%, and 70% of PPO during bipedal cycling [11,12,13]. Previous investigations have observed an inverse association between exercise intensity and power production asymmetry in trained cyclists [28,29,30]. Although the current data suggest that exercise intensity does not impact asymmetry levels in persons with MS, its role cannot be completely ruled out currently. Finally, the MS cohort in the current study had an average EDSS of 1.87 ± 1.09 (minimal impairment), indicating a lower disability level compared to the participants of previous investigations who had an average EDSS of 2.6 ± 1.6 (mild to moderate impairment) [12,13]. It can be speculated that asymmetry may not become prominent until higher levels of disability are reached.

Previous investigations examining asymmetries in muscle function have utilized percent differences between contralateral limbs in outcome measures to evaluate the presence of asymmetry. A percent difference between contralateral limbs in muscle function that is ≥10% has been previously defined to indicate the presence of asymmetry [14,24,25]. Although a statistically significant difference in asymmetry between groups was not present in the current study, the group average for the PwMS group exhibited asymmetry values ≥10% for power and force at 50%, 60%, and 70% of PPO (Figure 1). The group average for the Non-MS group did not exhibit asymmetry levels that exceeded 10% for either power or force at any of the measured exercise intensities. Additionally, PwMS had a higher percentage of the sample that reached the ≥10% threshold for both variables at all three exercise intensities compared to the Non-MS group (Figure 4). The levels of asymmetry in power and force production observed in the current study are similar to those previously reported for power production during unilateral cycling in PwMS (10–28%) [11]. It has been argued previously that statistical analysis in nonhomogeneous clinical populations may mask clinically relevant differences, and an alternative analysis may provide additional insight [31]. Assessing asymmetry based on a defined threshold may provide better insight than traditional analysis when observing persons with MS. Future research is needed to determine a clinically meaningful level of asymmetry in order to provide guidance for future analysis.

Both group averages for MVC strength exceeded the 10% threshold for asymmetry, with a greater portion of the Non-MS group exceeding the threshold than the PwMS group (Figure 3). The reason for this is unclear, but it could be speculated that greater between-limb differences in the body composition of the lower limbs could play a role. The Non-MS group had greater between-limb differences in fat mass, lean mass, and fat % for the lower limbs compared to the PwMS with small to large effective sizes observed, although these differences were not statistically different from the PwMS. The ability of muscles to generate force is related to measures of muscle cross-sectional area and volume [32]. The between-limb differences in body composition measures may have contributed to the high levels and prevalence of asymmetry seen in the Non-MS group.

Efficiency in pedaling during cycling involves the application of forces to the pedals in a manner that allows for the greatest translation of power into forward propulsion [33]. Due to limitations in equipment, the current study was only able to calculate efficiency in a manner that took into account the application of forces and translation into propulsive power of both pedals in combination with each other. However, this manner of application still has the potential to indicate imbalances in performance between the lower limbs. Higher levels of asymmetry in force production during cycling have been observed to induce lower levels of pedaling efficiency [33,34]. The current study did not observe any significant group × intensity interactions for pedaling efficiency, but a main effect for exercise intensity was present with levels of efficiency increasing as exercise intensity increased. This phenomenon has been observed in previous research with cyclists, and has been speculated to be related to improved muscular recruitment strategies resulting in improved force application and the minimization of the development of fatigue [33,35]. The improvement in efficiency in the current study is an interesting finding, as it has been documented that PwMS can experience a transient worsening of disease symptoms, as indicated by impairment in central motor conduction time and cortical excitability related to increases in core temperature during vigorous physical exertion [36,37,38]. The increase in core temperature impairs central motor conduction time and cortical excitability as a result of slowed or blocked conduction in demyelinated lesions in the central nervous system [38]. The observed improvement in efficiency with increasing exercise intensity in the current study may be due to the lower disability status within the current sample. The previously mentioned transient worsening symptoms may not have reached a degree that impaired the participants’ performance.

Correlation analysis was run between the percent asymmetry in power, force, and efficiency at each recorded exercise intensity and walking capacity. Interestingly, no significant correlations were present between cycling variables and performance on walking tasks when groups were combined or when analyzed with PwMS alone. Previous investigations examining the impact of asymmetry on walking performance in persons with MS have reported mixed results [13,14,39]. Larson et al. and Sandroff et al. both found that higher levels of asymmetry in PPO and knee extensor strength, respectively, were associated with lower performance walking performance [13,39]. However, Proessl et al. did not detect a significant association between knee extensor strength asymmetry and walking capacity in PwMS [14]. The results of the current study are in agreement with the results reported by Proessl et al. in that measures of asymmetry did not significantly relate to walking performance [14]. This is an interesting finding, as the levels of asymmetry in the current study are similar to those previously reported. Perhaps asymmetry does not play a significant role in walking performance until higher levels of disability are reached, as indicated by reductions in ambulation. Future research is needed to examine the association between measurements of asymmetry in various variables associated with lower extremity performance across a wide spectrum of ambulation capabilities to establish any associations.

We must acknowledge that our study is not without limitations. First, the current sample size is small, and the distribution of males and females is not representative of the MS population. Additionally, during the submaximal ICTs, the progressive nature of the workload may have allowed for development fatigue in a manner that may have influenced the asymmetry measures. Future studies investigating asymmetry should consider testing in a manner that reduces the accumulation of fatigue. Additionally, only assessing the strength asymmetry of the knee extensors may have inhibited the ability to determine the impact of asymmetry on walking capacity, as knee flexor strength has been shown to be a greater contributor to walking capacity in persons with MS [40]. Despite these limitations, the current authors feel that the findings of this study provide valuable insight to the area of asymmetries in muscle function in PwMS by being the first study to assess asymmetry in a natural bipedal movement.

## 5. Conclusions

In conclusion, the current study is the first study that allows for researchers to observe and analyze asymmetry between the lower limbs during a bipedal movement during submaximal exercise, rather than unilaterally and at maximal effort. The current study did not find any statistical difference in levels of asymmetry between PwMS and Non-MS in regard to power and force at 50%, 60%, and 70% of PPO during cycling and for knee extensor strength. Additionally, no statistically significant differences in efficiency during cycling, walking performance, and the MVC strength asymmetry score of the knee extensors were detected between groups. Also, measures of asymmetry and efficiency did not exhibit a significant association with walking performance in PwMS. Although the percent difference for asymmetry in power and force were not statistically different, the group average for PwMS displayed asymmetry levels above the established 10% threshold, indicating significant asymmetry for cycling variables, while the Non-MS group did not. Additionally, a greater portion of the PwMS sample exceeded the 10% threshold for asymmetry for power and force asymmetry than the Non-MS group. However, a greater portion of the Non-MS group exceed the 10% threshold for MVC strength asymmetry. This may be explained by greater between-limb differences in the body composition measures of the lower limbs. The current authors feel that these results provide additional support for the use of clinically meaningful differences when assessing asymmetry in this population, rather than relying solely on traditional statistical analyses. Future research is needed to establish a meaningful threshold for asymmetry specific to persons with MS. The current authors also speculate that, based on the findings of the current study and previous investigations, the impact of asymmetry may be dependent on ambulation capacity, and future research is needed to establish this association.

## Figures and Tables

**Table 1:** Participant characteristics.

Variable	PwMS (*n* = 8)	Non-MS (*n* = 6)	*p*	*d*
Age (yrs)	45.0 ± 12.1	45.5 ± 9.0	0.93	0.05
Height (cm)	174.0 ± 4.5	174 ± 10.4	0.93	0.00
Body Mass (kg)	94.5 ± 17.7	80.0 ± 6.2	0.79	1.09
Body Mass Index (kg/m^2^)	30.7 ± 6.2	26.6 ± 4.0	0.18	0.79
Body Fat (%)	41.1 ± 7.0	32.8 ± 14.2	0.17	0.74
Lean Mass (kg)	51.6 ± 6.6	51.8 ± 9.4	0.95	0.02
Fat Mass (kg)	37.0 ± 11.9	25.7 ± 11.9	0.10	0.95
Physical Activity (min/wk)	206.0 ± 180.0	260.0 ± 129.0	0.55	0.34
EDSS	1.9 ± 1.1	N/A	N/A	

Data are mean ± SD. Cohen’s *d* = effects sizes, EDSS = expanded disability status scale, PwMS = persons with multiple sclerosis.

**Table 2:** Difference between lower limbs for lean and fat mass.

Variable	PwMS Δ	Non-MS Δ	*p*	*d*
Lean Mass (kg)	0.15 ± 0.21	0.22 ± 0.15	0.48	0.38
Fat Mass (kg)	0.08 ± 0.11	0.13 ± 0.13	0.49	0.41
Lower-Limb Fat (%)	0.00 ± 0.00	0.02 ± 0.04	0.26	0.71

Data are mean ± SD. Cohen’s *d* = effect sizes.

**Table 3:** Physiological variables during the initial incremental cycling test.

Variable	PwMS	Non-MS	*p*	*d*
VO_2_peak (mL/kg/min)	24.0 ± 7.8	28.0 ± 10.8	0.43	0.42
Max Heart Rate (bpm)	157.2 ± 23.1	158.1 ± 25.5	0.95	0.04
Peak Power Output (W)	148.0 ± 39.5	168.0 ± 70.0	0.52	0.35

Data are mean ± SD. Cohen’s *d* = effect sizes, VO_2_peak = peak oxygen consumption.

**Table 4:** Variables from the submaximal incremental cycling test.

Variable	Group	50% PPO	60% PPO	70% PPO
Abs. Diff Power (W)	PwMS	12.7 ± 11.2	11.4 ± 9.9	12.2 ± 8.6
	Non-MS	4.88 ± 5.3	5.5 ± 4.7	3.5 ± 3.7
	*d*	0.89	0.76	1.3
% Diff Power (%)	PwMS	20.1 ± 18.6	15.5 ± 14.8	13.9 ± 12.1
	Non-MS	6.1 ± 5.4	5.4 ± 3.7	3.4 ± 4.0
	*d*	1.02	0.93	1.16
Abs. Diff Force (N·m)	MS	7.8 ± 6.9	5.7 ± 3.7	5.9 ± 5.2
	Non-MS	3.2 ± 3.4	4.0 ± 2.1	3.8 ± 1.7
	*d*	0.83	0.56	0.52
% Diff Force (%)	PwMS	16.4 ± 13.3	12.0 ± 7.8	10.4 ± 8.1
	Non-MS	7.1 ± 6.5	8.7 ± 4.6	8.0 ± 4.3
	*d*	0.88	0.51	0.36
Efficiency (%)	PwMS	56.4 ± 11.3	61.8 ± 12.7	67.2 ± 13.7
	Non-MS	62.6 ± 13.2	67.9 ± 12.2	74.5 ± 11.6
	*d*	0.50	0.49	0.58

Data are mean ± SD. Cohen’s *d* = effects sizes. Abs. Diff = absolute difference between lower limbs, % Diff = percent difference between lower limbs, PPO = peak power output.

**Table 5:** Correlation coefficients between walking performance and physiological variables from the submaximal increment cycling test.

Intensity	Variable	PwMS			Non-MS		
		% Diff Power	% Diff Force	Efficiency	% Diff Power	% Diff Force	Efficiency
50% PPO	T25FW	−0.34	−0.39	0.28	−0.21	0.12	−0.81 *
	6MWT	0.23	0.26	−0.39	0.07	0.59	0.77
60% PPO	T25FW	−0.31	−0.23	0.42	0.47	0.54	−0.83 *
	6MWT	0.05	−0.15	−0.46	0.47	−0.12	0.78
70% PPO	T25FW	−0.34	−0.17	0.40	−0.15	−0.84 *	−0.87 *
	6MWT	0.06	−0.29	−0.46	0.18	−0.58	0.73

Pearson’s product moment correlation coefficient reported. T25FW = Timed 25-Foot Walk; 6MWT = 6-Min Walk Test; % Diff = percent difference between lower limbs. * *p* ≤ 0.05 indicates significant correlation.

**Table 6:** Correlations coefficients between walking performance and knee extensor strength asymmetry scores.

Variable	KE Strength Asymmetry Score	
	PwMS	Non-MS
T25FW	−0.19	−0.18
6MWT	−0.27	0.02

Pearson’s product moment correlation coefficient reported. T25FW = Timed 25-foot Walk; 6MWT = 6-Min Walk Test; KE = knee extensor.

## References

[1] Mahad D.H., Trapp B.D., Lassmann H. (2015). Pathological mechanisms in progressive multiple sclerosis. Lancet Neurol..

[2] Carroll C.C., Gallagher P.M., Seidle M.E., Trappe S.W. (2005). Skeletal muscle characteristics of people with multiple sclerosis. Arch. Phys. Med. Rehabil..

[3] Chung L.H., Remelius J.G., Van R.E., Kent-Braun J.A. (2008). Leg power asymmetry and postural control in women with multiple sclerosis. Med. Sci. Sports Exerc..

[4] De Haan A., de Ruiter C.J., van der Woude L.H., Jongen P.J. (2000). Contractile properties and fatigue of quadriceps muscles in multiple sclerosis. Muscle Nerve.

[5] Garner D.J., Widrick J.J. (2003). Cross-bridge mechanisms of muscle weakness in multiple sclerosis. Muscle Nerve Med..

[6] Kent-Braun J., Ng A., Castro M., Weiner M., Gelinas D., Dudley G., Miller R. (1997). Strength, skeletal muscle composition, and enzyme activity in multiple sclerosis. J. Appl. Physiol..

[7] Ng A., Miller R., Gelinas D., Kent-Braun J. (2004). Functional relationships of central and peripheral muscle alterations in multiple sclerosis. Muscle Nerve.

[8] Wens I., Dalgas U., Vandenabeele F., Krekels M., Grevendonk L., Eijnde B.O. (2014). Multiple sclerosis affects skeletal muscle characteristics. PLoS ONE.

[9] Klaren R.E., Sandroff B.M., Fernhall B., Motl R.W. (2016). Comprehensive profile of cardiopulmonary exercise testing in ambulatory persons with multiple sclerosis. Sports Med..

[10] Goverover Y., Chiaravalloti N., DeLuca J. (2016). Brief International Cognitive Assessment for Multiple Sclerosis (BICAMS) and performance of everyday life tasks: Actual reality. Mult. Scler. J..

[11] White L.J., Dressendorfer R.H. (2005). Factors limiting maximal oxygen uptake in exertional monoparesis. Mult. Scler. J..

[12] Larson R.D., McCully K.K., Larson D.J., Pryor W.M., White L.J. (2014). Lower-limb performance disparities: Implications for exercise prescription in multiple sclerosis. J. Rehabil. Res. Dev..

[13] Larson R.D., McCully K.K., Larson D.J., Pryor W.M., White L.J. (2013). Bilateral differences in lower-limb performance in individuals with multiple sclerosis. J. Rehabil. Res. Dev..

[14] Proessl F., Ketelhut N.B., Rudroff T. (2018). No association of leg strength asymmetry with walking ability, fatigability, and fatigue in multiple sclerosis. Int. J. Rehabil. Res..

[15] Motl R.W., Pilutti L.A. (2012). The benefits of exercise training in multiple sclerosis. Nat. Rev. Neurol..

[16] Mills R., Young C., Pallant J., Tennant A. (2010). Rasch analysis of the Modified Fatigue Impact Scale (MFIS) in multiple sclerosis. J. Neurol. Neurosurg. Psychiatry.

[17] Schwid S.R., Covington M., Segal B.M., Goodman A.D. (2002). Fatigue in multiple sclerosis: Current understanding and future directions. J. Rehabil. Res. Dev..

[18] Larson R., Cantrell G., Ade C., Farrell J., Lantis D., Barton M., Laron D. (2015). Physiological responses to two distinct maximal cardiorespiratory exercise protocols. Int. J. Sport Exerc. Med..

[19] Borg G. (1971). The perception of physical performance. Front. Fit..

[20] Nolan P., Beaven M., Dalleck L. (2014). Comparison of intensities and rest periods for VO_2_max verification testing procedures. Int. J. Sports Med..

[21] Fischer J., Rudick R., Cutter G., Reingold S. (1999). The Multiple Sclerosis Functional Composite measure (MSFC): An integrated approach to MS clinical outcome assessment. National MS Society Clinical Outcomes Assessment Task Force (Force, N.M.S.C.O.A.T). Mult. Scler. J..

[22] Savci S., Inal-Ince D., Arikan H., Guclu-Gunduz A., Cetisli-Korkmaz N., Armutlu K., Karabudak R. (2005). Six-minute walk distance as a measure of functional exercise capacity in multiple sclerosis. Disabil. Rehabil..

[23] Hart S., Gabbard C. (1996). Brief communication: Bilateral footedness and task complexity. Int. J. Neurosci..

[24] Carpes F.P., Rossato M., Mota C.B., Faria I.E. (2006). Bilateral pedaling asymmetry during a simulated 40 km cycling time-trial. Med. Sci. Sports Exerc..

[25] Ithurburn M.P., Paterno M.V., Ford K.R., Hewett T.E., Schmitt L.C. (2015). Young athletes with quadriceps femoris strength asymmetry at return to sport after anterior cruciate ligament reconstruction demonstrate asymmetric single-leg drop-landing mechanics. Am. J. Sports Med..

[26] Cohen J. (1988). Statistical Power Analysis for the Behavioral Sciences.

[27] Olejnik S., Algina J. (2003). Generalized eta and omega squared statistics: Measures of effect size for some common research designs. Psychol. Methods.

[28] Carpes F.P., Mota C.B., Faria I.E. (2010). On the bilateral asymmetry during running and cycling—A review considering leg preference. Phys. Ther. Sport.

[29] Carpes F., Rossato M., Faria I., Mota C.B. (2007). During a simulated 40-km cycling time-trial. J. Sports Med. Phys. Fit..

[30] Carpes F.P., Rossato M., Faria I.E., Mota C.B. (2008). During an incremental exercise cyclists improve bilateral pedaling symmetry. Braz. J. Biomotricity.

[31] Revicki D.A., Cella D., Hays R.D., Sloan J.A., Lenderking W.R., Aaronson N.K. (2006). Responsiveness and minimal important differences for patient reported outcomes. Health Qual. Life Outcomes.

[32] Fukunaga T., Miyatani M., Tachi M., Kouzaki M., Kawakami Y., Kanehisa H. (2001). Muscle volume is a major determinant of joint torque in humans. Acta Physiol. Scand..

[33] Rossato M., Bini R., Carpes F., Diefenthaeler F., Moro A. (2008). Cadence and workload effects on pedaling technique of well-trained cyclists. Int. J. Sports Med..

[34] Bini R.R., Hume P.A. (2015). Relationship between pedal force asymmetry and performance in cycling time trial. J. Sports Med. Phys. Fit..

[35] Zameziati K., Mornieux G., Rouffet D., Belli A. (2006). Relationship between the increase of effectiveness indexes and the increase of muscular efficiency with cycling power. Eur. J. Appl. Physiol..

[36] Giesser B.S. (2015). Exercise in the management of persons with multiple sclerosis. Ther. Adv. Neurol. Disord..

[37] Marino F.E. (2009). Heat reactions in multiple sclerosis: An overlooked paradigm in the study of comparative fatigue. Int. J. Hyperth..

[38] Davis M.P., Walsh D. (2010). Mechanisms of fatigue. J. Support. Oncol..

[39] Sandroff B.M., Sosnoff J.J., Motl R.W. (2013). Physical fitness, walking performance, and gait in multiple sclerosis. J. Neurol. Sci..

[40] Mañago M.M., Hebert J.R., Kittelson J., Schenkman M. (2018). Contributions of Ankle, Knee, Hip, and Trunk Muscle Function to Gait Performance in People with Multiple Sclerosis: A Cross-Sectional Analysis. Phys. Ther..

